# Transmission and control pressure analysis of the COVID-19 epidemic situation using multisource spatio-temporal big data

**DOI:** 10.1371/journal.pone.0249145

**Published:** 2021-03-29

**Authors:** Fangxiong Wang, Ziqian Tan, Zaihui Yu, Siqi Yao, Changfeng Guo

**Affiliations:** School of Geography, Liaoning Normal University, Dalian, China; Institute for Advanced Sustainability Studies, GERMANY

## Abstract

Taking the Guangdong-Hong Kong-Macao Greater Bay Area as the research area, this paper used OD cluster analysis based on Baidu migration data from January 11 to January 25 (before the sealing-off of Wuhan) and concluded that there is a significant correlation 1the migration level from Wuhan to the GBA and the epidemic severity index. This paper also analyzed the migration levels and diffusivity of the outer and inner cities of the GBA. Lastly, four evaluation indexes were selected to research the possibility of work resumption and the rating of epidemic prevention and control through kernel density estimation. According to the study, the amount of migration depends on the geographical proximity, relationship and economic development of the source region, and the severity of the epidemic depends mainly on the migration volume and the severity of the epidemic in the source region. The epidemic risk is related not only to the severity of the epidemic in the source region but also to the degree of urban traffic development and the degree of urban openness. After the resumption of work, the pressure of epidemic prevention and control has been concentrated mainly in Shenzhen and Canton; the further away a region is from the core cities, the lower the pressure in that region. The mass migration of the population makes it difficult to control the epidemic effectively. The study of the relationship between migration volume, epidemic severity and epidemic risk is helpful to further analyze transmission types and predict the trends of the epidemic.

## Introduction

The World Health Organization (WHO) has named the novel coronavirus responsible for the pneumonia outbreak in Wuhan, China as "novel coronavirus 2019" (2019-NCOV) on January 12, 2020. On February 11, WHO announced the official name of the disease caused by novel coronavirus infection, "Covid-19". On the same day, the International Committee on the Classification of Viruses announced that it would name the disease-causing novel coronavirus "SARS-CoV-2". It is a novel virus with high infectiousness and mortality rate that is more harmful than the previous MERS [1–4]. The first COVID-19 case was confirmed in Wuhan in late 2019 [5]. The 2019-nCov virus was defined by the WHO as a "public health emergency of international concern" on January 30, 2020 [6, 7].

The first imported COVID-19 case was found in Shenzhen on January 19, 2020, in the Guangdong-Hong Kong-Macao Greater Bay Area, and the first confirmed COVID-19 patients were found in Hong Kong and Macao on January 22; both individuals were from Wuhan or had passed through Wuhan [8–10]. It can be inferred from such data that population migration is one of the important factors affecting the severity of the epidemic.

With the rapid development of information and communication technology (ICT), big data has been widely used in the field of public health [11–13]. After the outbreak of the epidemic, domestic and foreign scholars conducted in-depth studies on the regional epidemic situation based on big data, combined with mathematical models and spatial analysis, and confirmed cases in various regions. Zian Zhuang et al. [14] estimated the confirmed cases in Iran based on air migration data and the confirmed cases imported from Iran to the Middle East, with predictive accuracy of approximately 95%. Sarkodie Samuel Asumadu et al. [15] used controllable section correlation, endogeneity and unobserved heterogeneity as evaluation methods to research the 31 provinces/municipalities directly under the central Chinese government. The research shows that the attributable death and the confirmed COVID-19 cases appear to have a linear correlation, and the recovered cases and the confirmed cases appear to have a nonlinear relationship. Liu Zhang et al. [16] analyzed the spatial distribution pattern of the migration into Hubei Province from Wuhan before New Year’s Eve based on geospatial big data and proposed a crowd dynamic assessment model. Yang Zheng et al. [17] conducted statistical analysis of the infection rate for the cities that saw migration from Wuhan, based on the big data provided by Baidu for migration in the 12 days from 2020.1.10 to 2020.1.22 and the confirmed number of COVID-19 cases in 50 Chinese cities. Zhu Renjie et al. [18] studied and predicted 7 countries with severe epidemics from 2020.3.4 to 2020.4.4 based on the Susceptible Infected Recovered Model. This study showed that the United States and the United Kingdom had uncontrollable epidemics that needed to strengthen prevention efforts. Xu Xiaoko et al. [19] analyzed the population mobility before the sealing-off of Wuhan based on big migration data provided by Tencent and Baidu. Hua and Shaw summarized the response of COVID-19 in China in the first three months. Shaw and Wiki et al. analyzed the countermeasures and key methods adopted in Japan (clustering method) [20–22].

The above scholars have carried out studies on the temporal and spatial distribution of the epidemic situation, as well as the migration volume and incidence rate, based on big data and the status of confirmed cases to predict the future trends of the epidemic. Wuhan was selected as the center of most research areas to analyze the impact of the migration from Wuhan on the development of the epidemic situation. In addition, most of the existing studies focus on COVID-19 at the national level, but there remains a lack of detailed studies on smaller study areas. Since the development of epidemics always starts in cities, it is of great significance to study urban agglomerations to prevent and control epidemics [23, 24].

The GBA, which is internationally representative, is selected as our research area. The GBA is one of the city clusters with the strongest economic vitality and the most obvious advantages in China. It is also the region with the closest business contacts and personnel exchanges with foreign countries, and it has a dense transportation network. Due to the high mobility of personnel in this region, the epidemic there has had a high severity and a long duration, making it a representative research area in China. This study aims to analyze the correlation between the migration scale index and the number of confirmed cases in all cities in this area before the closure of Wuhan, establish a transmissibility model for the epidemic situation affected by population mobility within one month after the closure of Wuhan, and analyze the degree of risk presented by population mobility to the epidemic situation in the urban agglomeration. Combined with migration data and POI data related to population density, the epidemic pressure level model was established from the four evaluation factors of population distribution, transportation hub, daily services, and medical and health care to assess the prevention and control pressure of the epidemic. This article’s research significance lies in the use of the close ties among the migration scale index, the epidemic severity and the disease risk, which can be further analyzed through imported cases and transmission type. The use of the index of epidemic prevention and control pressure can provide a reference for the resumption of work and for future study and can also provide a data model of contingency plans for similar national events.

## Data

### General situation of the research area

The GBA comprises two special administrative regions, namely, Hong Kong and Macao, and nine cities in the Pearl River Delta (PRD), namely, Canton, Shenzhen, Foshan, Tungkun, Huizhou, Zhuhai, Chungshan, Kongmoon and Shiuhing. As an international scientific and technological innovation center with global influence, the GBA, together with the Tokyo Bay Area of Japan, the New York Bay Area of the United States and the San Francisco Bay Area, are the four bay areas in the world.

**Fig 1:**
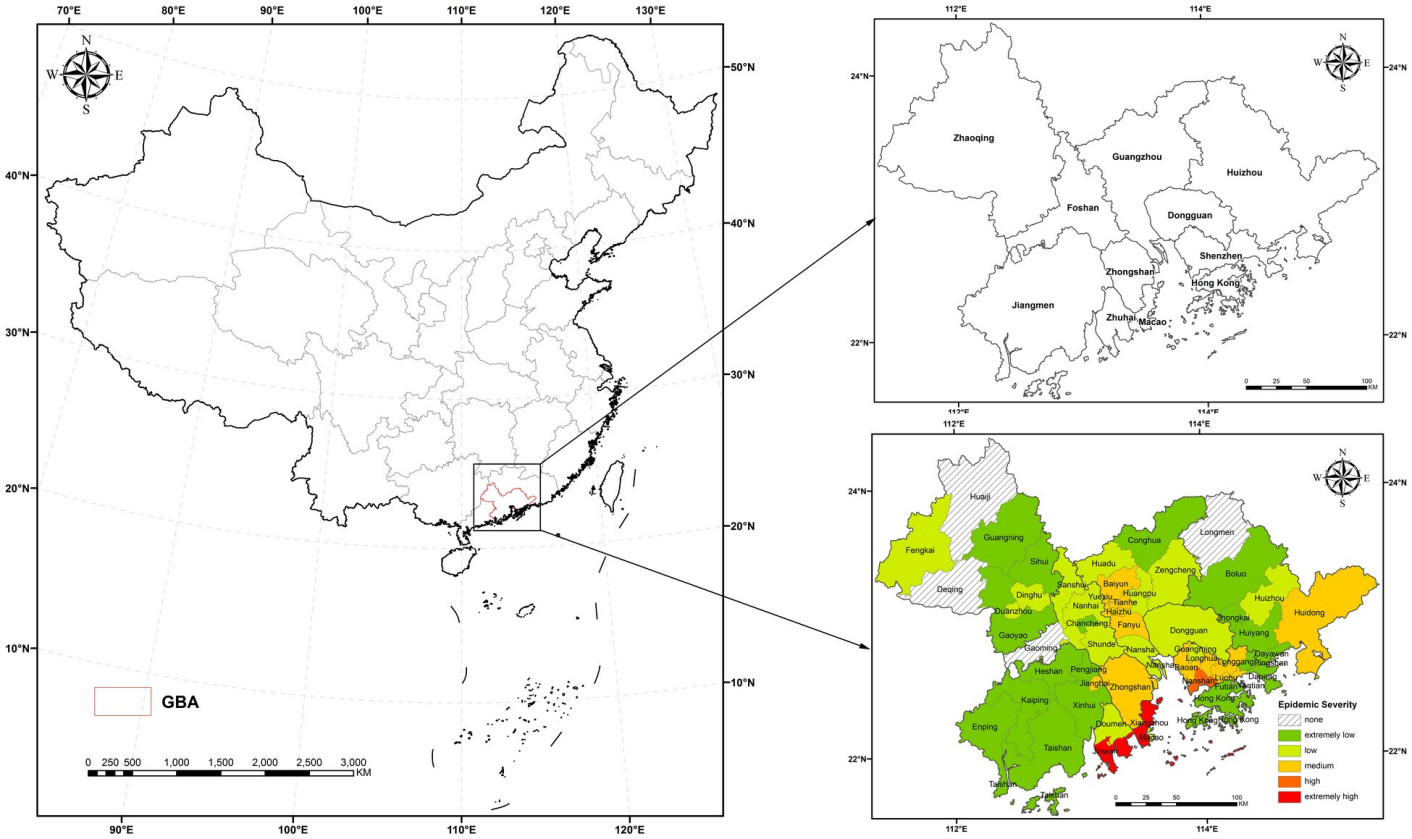


The GBA is a world-class city cluster with great economic vitality and international competitiveness. The region, located in the subtropical monsoon climate zone, is a high-quality environment suitable for living, working and traveling. By the end of 2018, the Greater Bay Area had a total resident population of approximately 70 million and a GDP of over 10 trillion yuan. As of February 22 this year, the region along the mainland and island coastline in this area has a severe epidemic situation, with the city of Zhuhai being the most severely affected (Fig 1).

### Data sources and processing

1. Baidu migration big data. These data are used to estimate the number of people flowing from Wuhan into prefecture-level cities in the GBA before the Spring Festival in 2020 and the crowd flow in prefecture-level cities in the study area after the Spring Festival. The data set comes from Baidu Map Smart Eye platform (http://qianxi.baidu.com/) and provides the proportion of the daily number of people moving in or out of other cities in each prefecture-level city and the total number of people moving in or out of the destination city. This paper obtained the proportion of the people who entered from Wuhan in each prefecture-level city in the GBA within 14 days before New Year’s Eve, 2020 and the proportion of the people who moved in and out among prefecture-level cities in the GBA to other cities within one month after the closure of Wuhan (S1–S3 Tables).
2. The resident population of the national district cities, as given by the population statistics at the end of 2018 from the provincial bureau of statistics released by the Statistical Yearbook [25–27], is used for modeling calculations in the real population. Considering the synchrony and the integrality between Tencent’s positioning big data and the resident population statistics at the city scale by the end of the year, this paper uses the data from the end of 2018 as mentioned above (S4 Table).
3. The cumulative number of confirmed COVID-19 cases and fever clinic data in prefecture-level cities and districts in the GBA were obtained to February 22, 2020, from the website of Guangdong Provincial Health Commission and the public releases of the prefectural health residents’ committees [28]. These data were used to analyze the severity of the epidemic in each city in the GBA and the correlation of severity with the population mobility at the prefectural level (S4 Table).
4. All the POI data in the evaluation factors are taken from the network electronic map, and the data points covering the entire research area were crawled on the Baidu map [29] through crawling technology, including 179,737 POI data points such as airports, railway stations, ports, bus stations, subway stations, hospitals, outpatient services, bazaars, supermarkets and shopping malls (S5 Table).

All the methods of collecting data sets in the manuscript are in accordance with the terms and conditions of the website.

## Methods

### Model of epidemic transmission force

OD data constitute a kind of trajectory data from the starting point to the end point that is directional but does not record specific trajectory path information. Migration data for population flow constitute one kind of OD data. Migration data contain attribute fields such as source name, destination name, migration date and migration volume. An OD flow graph is a visual analysis method that connects the starting place and destination with straight lines or curves, reflecting the OD flow through line thickness [30–35]. In this paper, the location information of the source and destination of the migration provided by the Baidu migration data is used to conduct clustering processing according to the time information of the migration data. Finally, the traffic map is projected onto the map through "node-link" visualization. The density of OD data is controlled by the classification method of natural break points to reflect the OD connection between cities and the volume of traffic.

Since the transmission force is not only generated by the population movement between cities, but also the influence of the population movement within cities, a single transmission force model cannot cover all the cases of population movement. Therefore, this paper proposes two models of epidemic transmission force for different population mobility.

#### Model of imported transmission force

Since the urban agglomeration is composed of several cities, the following two situations are divided into the following when studying the input propagation force:

1. The input communication brought by the migration of external cities outside the urban agglomeration into the urban agglomeration.
2. Imported transmission caused by population migration among cities within the urban agglomeration.

In essence, the migration of population from outside cities into urban agglomerations or from within urban agglomerations into each other is the migration of population between cities, and the transmission force brought by it is unity and input transmission force. With population migration data as the main index, the degree of connection between the emigrated cities and the cities in the urban agglomeration in a certain period of time was calculated, and the severity of the epidemic in the emigrated cities was taken as the influencing factor of the transmission capacity, so as to establish the index model of the imported transmission capacity of urban epidemic as follows:

$$T_{external}^{i}=\sum_{n=1}^{N}\left(\sum_{b}^{a}(M_{total}^{i}\times r_{n}^{i}\times S_{n}^{i}\right)),(i=1,2\ldots 11,n=1,2\ldots N)$$ (1)

$$S_{n}^{i}=D_{people}^{n}/P_{local}^{n}$$ (2)

$T_{external}^{i}$ is the epidemic transmission force index calculated by the external migration of the ith city in the urban agglomeration in the study area. a and b are the head and tail nodes of the study period, respectively, and N is the total number of cities that have moved into the ith city. $M_{total}^{i}$ is the total amount of the influx into city i on that day, and $r_{n}^{i}$ is the migration ratio of city n to city i in the urban agglomeration on the same day, that is, the migration ratio of the source area; therefore, $M_{total}^{i}\times r_{n}^{i}$ is the migration amount of city n to city i in the urban agglomeration.

Among these factors,$S_{n}^{i}$ is the severity of the epidemic in the external region on that day, $D_{people}^{n}$ is the number of confirmed cases in the city on that day, and $P_{local}^{n}$ is the number of urban registered population. The number of confirmed cases cannot be used directly as an indicator of the severity of the epidemic in the region due to the different populations in each city. Since the outbreak occurred during the Spring Festival, the total registered population in these cities is closer to the actual local population.

#### Model of dispersive transmission force

To study urban agglomeration in the cities brought about by the spread of invasive of internal population flow, the model for each city within the city travel intensity as the main index, and according to the city the disease severity as the transmission force of impact factor, calculated at a certain time period, the transmission force produced by the city travel outbreak, establish city dispersive transmission forces model is as follows:

$$T_{inner}^{i}=\sum_{b}^{a}\left(P_{i}/P_{local}\right)\times S_{i},(\text{i}=1,2\ldots 11)$$ (3)

$T_{inner}^{i}$ is the epidemic transmission power index calculated for the internal travel of the ith city in the urban agglomeration of the study area; among these factors, *P*_*i*_ is the visitor flow rate in the city, *P*_*i*_/*P*_*local*_ is the traveling intensity in the city, and *S*_*i*_ is the severity of the epidemic of the ith city.

### Model of epidemic prevention and control stress

In this study, factors related to population density were selected and classified into 4 categories of evaluation factors, which were then subdivided into 14 subclasses. Here, based on the epidemic prevention and control pressure level, the 4 categories of evaluation factors were used to analyze the POI data and screen the POI type of urban agglomeration related to the evaluation factors as subclasses of evaluation factors. Expert evaluation and the paired comparison method were used to compare the two kinds of POI data to provide a comprehensive rating evaluation [36, 37].

For POI point data, point density analysis is adopted and generally includes the quadrat density method and the kernel density method. The quadrat density method ignores the difference of density values at different positions inside the unit because the density values at all positions inside the unit are equal, leading to no continuity of density values between the units and large span values, which will affect subsequent analysis. Therefore, the kernel density method is adopted in this paper to solve these problems. The kernel density function is used to calculate the density of point elements in its surrounding neighborhood, and each point is fitted to a smooth cone surface. The core density value reflects that the distance affected by the distance from the center point decreases gradually, which accounts for the attenuation effect of point elements on the distance to the facilities and services affected in its neighborhood [38, 39].

The kernel density function can be calculated as follows:

$$f(s)=\sum_{n}^{i=1}\frac{1}{h^{2}}k(\frac{s-c_{i}}{h})$$ (4)

In Formula (4), f(s) is the kernel density calculation function at space position s; h is the distance attenuation threshold; n is the number of elements whose distance from position s is less than or equal to h; the k function represents the space weight function. The geometric meaning of this function equation is expressed as the density value reaching the maximum at the core point *c*_*i*_, and the kernel density value gradually decreasing with the increase of the distance affected by the core, until the distance from the core *c*_*i*_ reaches the threshold h, when the kernel density value drops to 0.

The choice of k(·) function exerts little influence on the density analysis because the parameter is based on the distance attenuation effect. The setting of search radius h is often based on POI data and the practical application environment, and there are many possible influencing factors, such as analysis scale and dispersion degree of POI points.

Based on the migration data and nuclear density data obtained above, the index frequency density constructed by Chi Jiao and the epidemic prevention and control level model mentioned by Li Gang in a lecture on epidemic prevention and control [40], and taking the urban agglomeration research area, district and county level as the research unit, the nuclear density superposition index and the epidemic prevention and control pressure index model are established as follows:

$$K_{i}=\sum_{n_{i}=1}^{N}k_{n_{i}}(i=1,2\ldots 14,n_{i}=1,2\ldots N)$$ (5)

$$M_{level}=\sum_{j=1}^{4}A_{j}\times w_{j}(j=1,2\ldots 4)$$ (6)

Among these factors, i represents the type of POI in the evaluation factor subclass, *n*_*i*_ represents the pixel points of type i in the research unit, n represents the total pixel numbers in the research unit, and $k_{n_{i}}$ represents the POI kernel density value corresponding to the pixel points of type i in the research unit. *K*_*i*_ represents the total nuclear density of POI i within a cell.

*M*_*level*_ represents the epidemic prevention and control pressure index, j represents the types of evaluation factors, k_j_ represents the density value of population distribution, transportation hub, health care and life services after homogenization, and *w*_*j*_ represents the weight of evaluation factors A1, A2, A3 and A4, respectively.

## Results

### Imported transmission force of the urban agglomeration

#### The imported transmission force before city closure in Wuhan

The first case of COVID-19 in China was confirmed on December 8, 2019 in Wuhan. This area evolved into a gathering place in the early stage of the epidemic, which would erupt in China on approximately January 20, 2020. With the Spring Festival on January 25 as the node, the period from January 11 to January 25 is the first peak period of the Spring Festival travel rush, which is the largest, most widely distributed and most difficult-to-control event of population migration in China. During the Spring Festival travel season, the epidemic was aggravated by population mobility and the spread of the virus, which spread and worsened all over the country, with the city of Wuhan as the center. According to the latest “COVID-19 diagnosis and treatment plan”, based on the current epidemiological investigation, the incubation period of the coronavirus is approximately 1–14 days. Therefore, we selected the first day of the closure in Wuhan as the deadline for the data points to obtain the migration volume from Wuhan to each urban agglomeration in the GBA within the period from January 11 to January 24 to analyze the correlation between the migration volume and the epidemic severity index.

**Fig 2:**
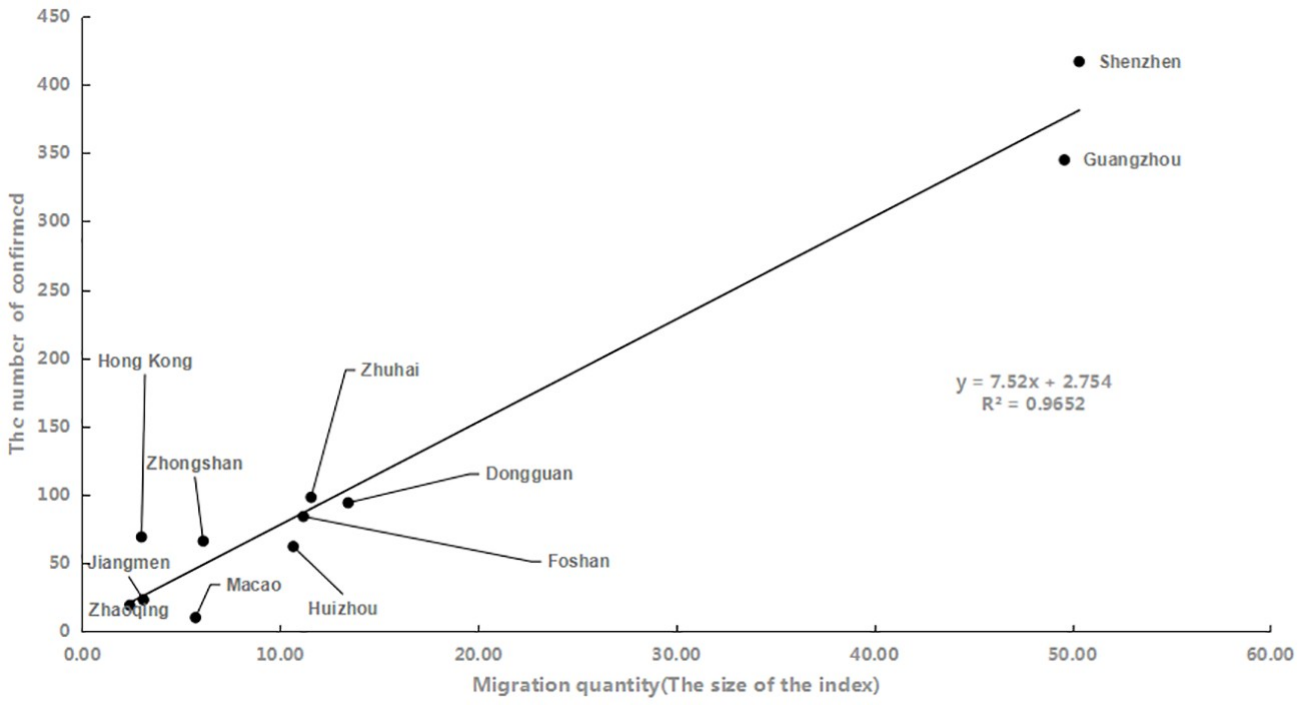


To February 22 (a month after the Wuhan sealing), the agglomeration of the GBA saw a total of 1287 confirmed cases. The number of confirmed cases in each city in the urban agglomeration from 14 days before the closure of Wuhan was positively correlated with the amount of migration from Wuhan. As shown in Fig 2, R^2^ is 0.96, and the correlation coefficient of 0.98; the increment of independent variable migration, the dependent variable number increase, and diagnosis were significantly related. There were 417 confirmed cases in Shenzhen and 435 in Guangzhou originating from Wuhan, accounting, respectively, for 32% and 27% of the total confirmed cases. Zhuhai, Dongguan, Foshan and Hong Kong have 5%-10% of confirmed cases, while the rest of the cities have less than 5%.

**Fig 3:**
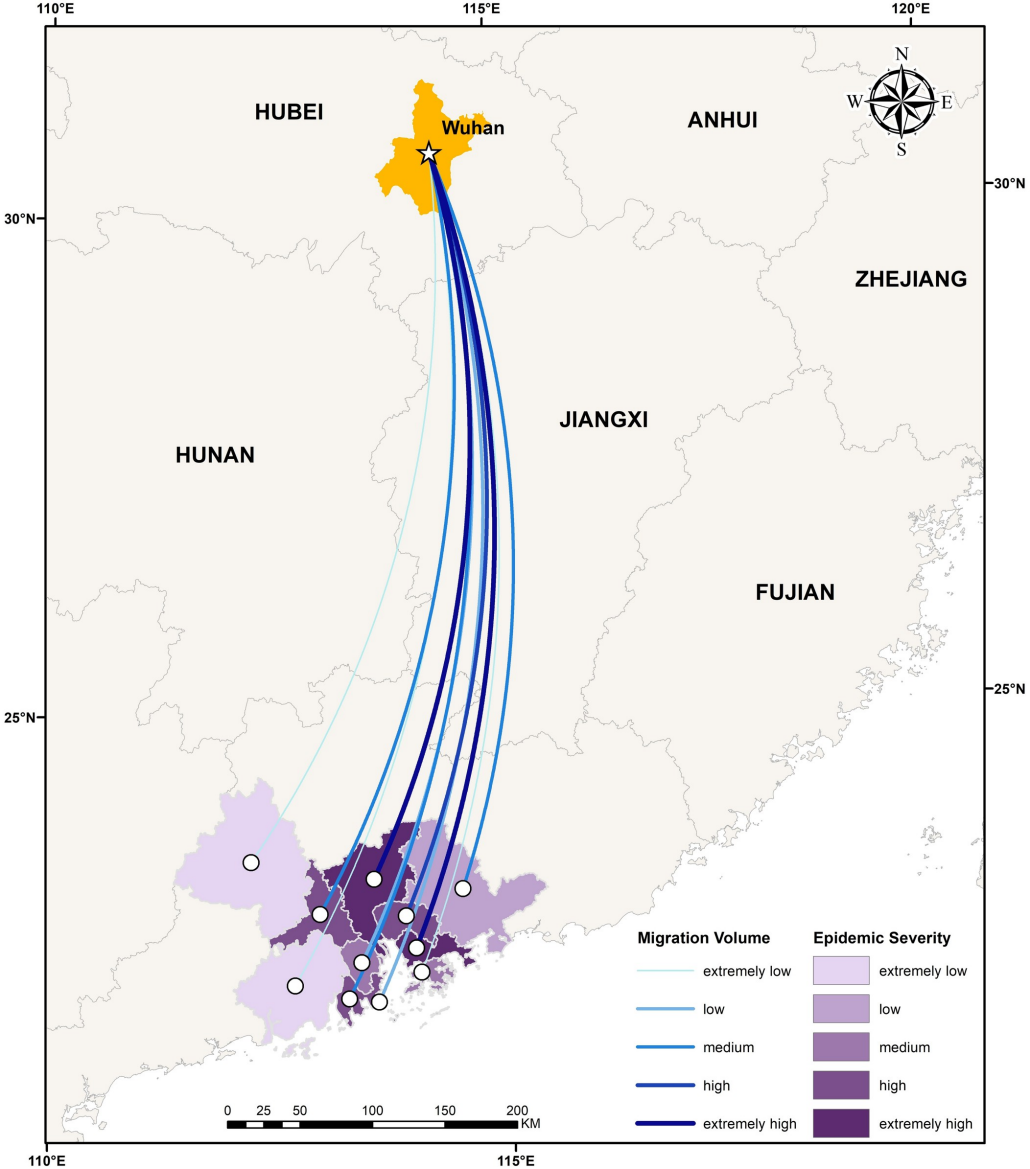


The total amount of migration from Wuhan to the cities in the urban agglomeration in the period from January 11 through the solstice to January 24 was used in the OD flow chart to show the OD relationship between cities, with the color and thickness of the line elements used to represent the flow size. According to the analysis in Fig 3, the migration is divided into five grades: extremely low, low, medium, high and extremely high. Shenzhen, Guangzhou and Dongguan had the top three largest numbers of people moving from Wuhan, which is consistent with the "reverse Spring Festival travel" rule in recent years. The rest areas, centering on Shenzhen, Guangzhou and Dongguan, show a decreasing trend with the increase of distance. The epidemic severity index (epidemic severity index = number of confirmed cases/registered population) is the same as the classification rules of migration. With Guangzhou and Shenzhen as the central axis, the epidemic severity index decreases from east to west, forming a low-high-low epidemic severity index model from east to west.

Before the closure of Wuhan, the population migration index from Wuhan to cities in the urban agglomeration was significantly correlated with the number of confirmed cases in the study area, so it was speculated that the population migration index could be used as an important indicator to evaluate the epidemic situation. An analysis of imported sexually transmitted diseases after the closure of Wuhan selected the 31 days from January 23 to February 22 as the research period, extracted the source areas of migration in cities in the urban agglomeration as the research object, and analyzed the migration index between cities and the epidemic risk caused by the epidemic degree.

#### The imported transmission force of external cities

Twenty major external sources of migration in cities in the urban agglomeration are selected as the starting places. An OD flow map is created with the cities in the urban agglomeration as destinations, and the OD flow is the sum of the migrations across 31 days.

**Fig 4:**
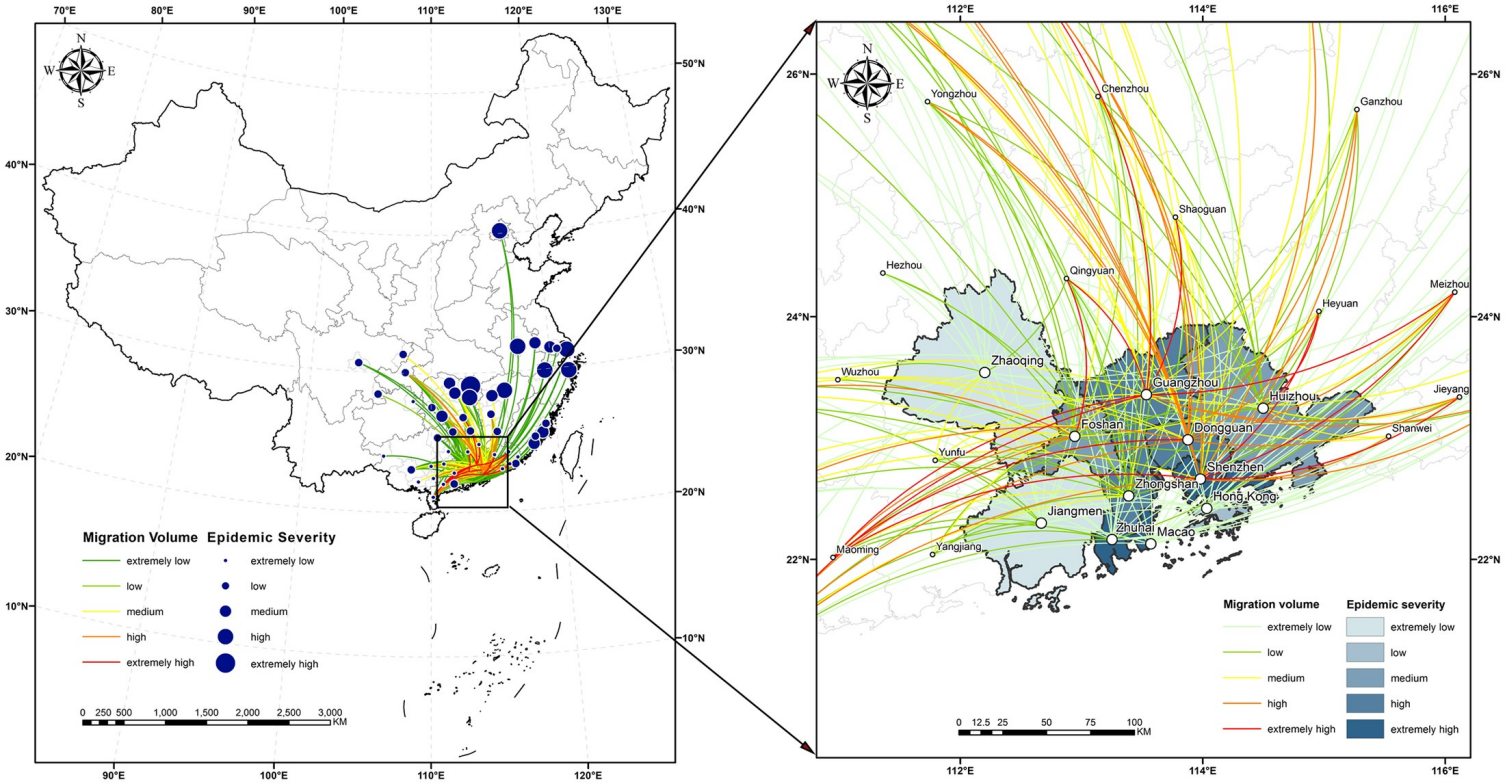


As shown in Fig 4, Shenzhen and Zhuhai had been severely affected by the epidemic by 24:00 on February 22. Seven of Shenzhen’s cities of origin were from Hunan Province, which was relatively severely affected by the epidemic: Shaoyang, Hengyang, Yongzhou, Changde, Chenzhou, Huaihua and Yueyang. The migration index of Zhuhai accounts for only 3% of the total migration volume of the entire city cluster. The reason for the severity of the epidemic is that it is close to the South China Sea and has close economic and trade contact with overseas countries. In the urban agglomeration, the city with the largest migration volume is Guangzhou, with Shenzhen in second place. The total migration volume of these two cities accounts for 47% of the total migration volume of the entire urban agglomeration. The main migration city of Guangzhou is outside the study area of Guangdong Province. In addition, the Chongqing municipality, the Guangxi Zhuang Autonomous Region and Central Guigang and Ganzhou in Shanxi Province, which are adjacent to Guangdong Province, have seen a large influx of migrants. Although number of migrants is large, the epidemic degree in Guangzhou is medium because of the low epidemic degree of the sources. Dongguan and Zhongshan have the same epidemic level as Guangzhou due to the small number of registered residents. The number of migrants in Foshan and Huizhou was much higher than that in Jiangmen and Zhaoqing, so the epidemic degree in Foshan and Huizhou was low, while that in Jiangmen and Zhaoqing was extremely low.

The immigration population of Hong Kong and Macao was significantly lower than that of the Pearl River Delta, accounting for 0.44% and 0.21% of the total migration of the study area, respectively. Hong Kong and Macau are located mainly in the Pearl River Delta and the economically developed Yangtze River Delta. Seven cities from the Pearl River Delta, including Shanwei, Qingyuan and Meizhou, and seven from the Yangtze River Delta, including Shanghai, Beijing and Hangzhou, made the top 20 for migration to Hong Kong. Five cities from the Pearl River Delta and eight from the Yangtze River Delta are in Macao’s top 20 for migration. In addition, the non-Pearl River Delta and Yangtze River Delta cocities with movement to Macao and Hong Kong were mostly cities with high GDP, such as the Chongqing municipality, Quanzhou city, Chengdu city and Xiamen city. Hong Kong and Macao are economically active and densely populated, and the epidemic degree of these source regions is significantly higher than that of other cities in the study area. However, the migration volume is very small, resulting in a low epidemic degree.

**Fig 5:**
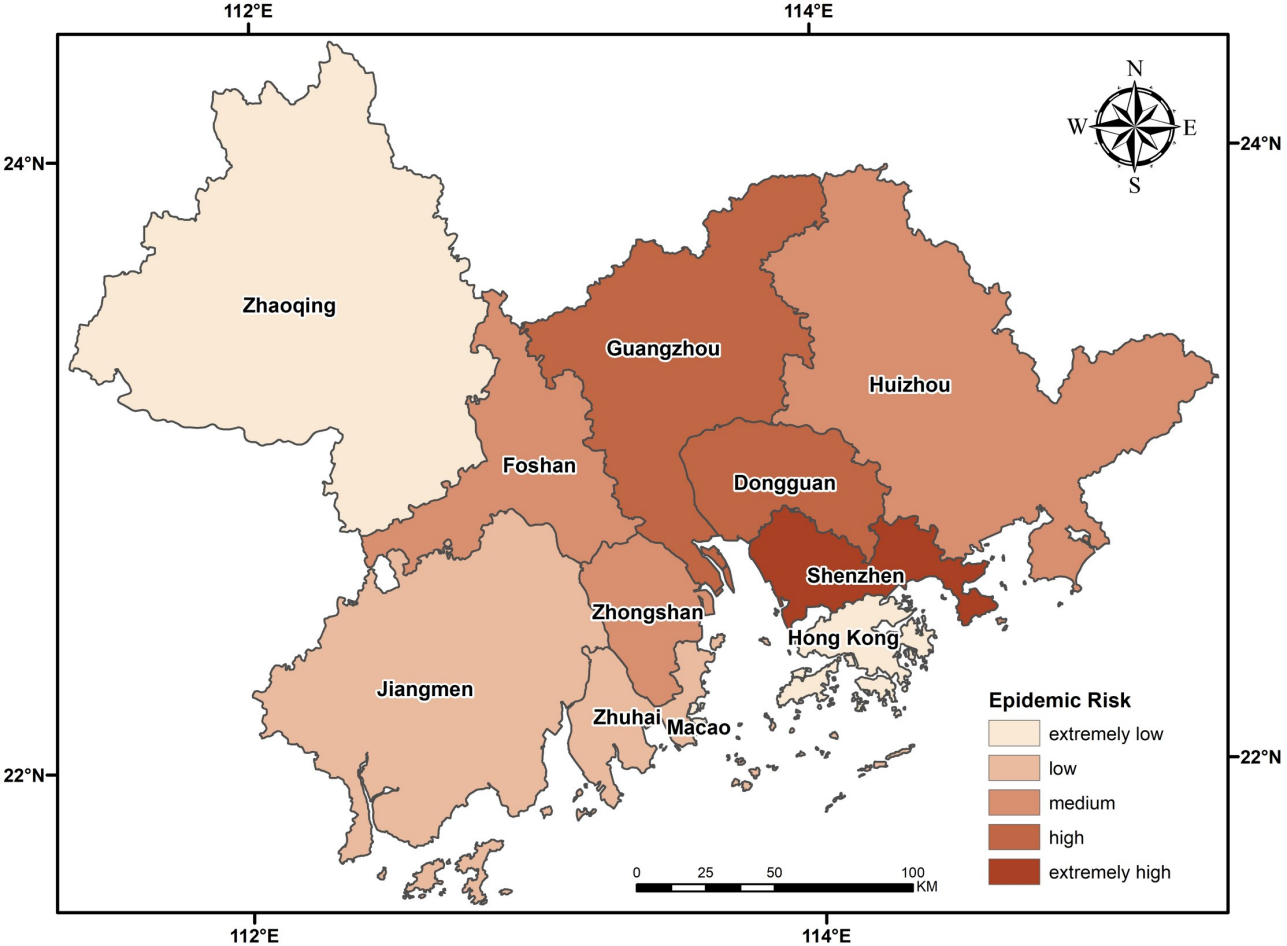


The calculated migration data and the epidemic severity index of each city were input into the epidemic transmission force model. a and b were set as 1.23 and 2.22, and the epidemic risk level chart of the migration of external cities was obtained. As shown in Fig 5, the epidemic risk of all cities in the urban agglomeration is centered on Guangzhou, Shenzhen and Dongguan, three severe epidemic areas. The further away from the center an area is, the more the epidemic risk index decreases gradually in a circular pattern. The epidemic risk indexes of Shenzhen, Dongguan and Guangzhou are much higher than those of other regions, with the risk index of Shenzhen accounting for 46% of the total index. The first reason for such large numbers is that the migration volume of these three cities is in the top three and is much higher than that of Foshan, the fourth. The scale indexes of Guangzhou, Shenzhen and Dongguan, respectively, account for 24%, 23% and 20% of the total migration volume. The second reason is that the individuals moving into the urban agglomeration come mainly from other cities in Guangdong Province and Hunan Province, which has a relatively severe epidemic situation. Among the 50 cities with individuals moving into the urban agglomeration, Yueyang city in Hunan Province has the highest epidemic situation, followed by Nanchang and Changsha, with relatively severe epidemic situations. The epidemic risk indexes of Foshan, Huizhou, Zhongshan, Zhuhai, Jiangmen and Zhaoqing decreased with the decrease of migration volume. Hong Kong and Macao have a small influx, but the higher risk of COVID-19 there than in Zhaoqing is due to the serious risk of COVID-19 in the source region.

#### The imported transmission force inside the urban agglomeration

An OD flow map was created with each city in the urban agglomeration as the starting place and destination, and the OD flow was the sum of the migrations across 31 days.

**Fig 6:**
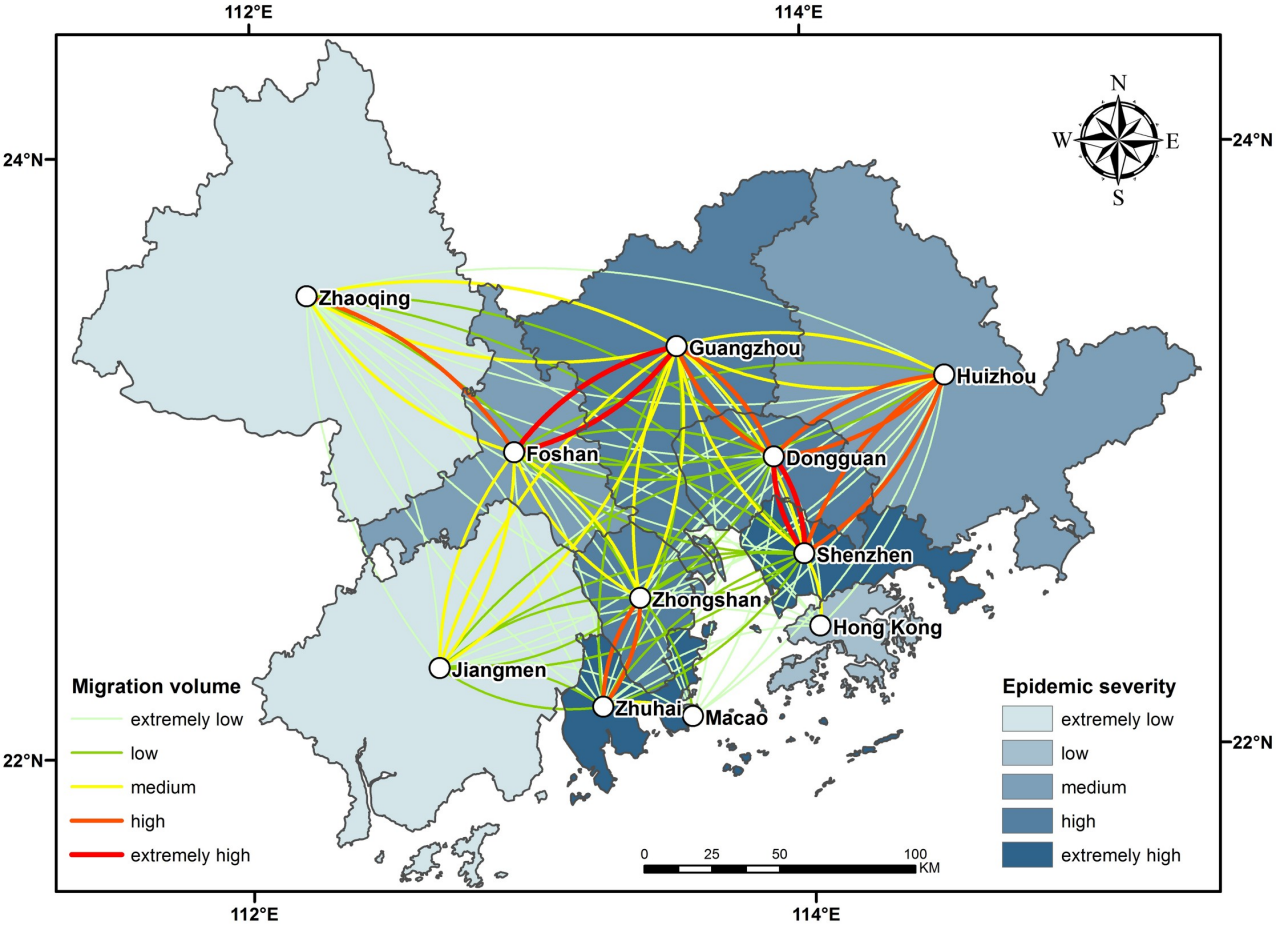


As shown in Fig 6, the population mobility tend to move to the prosperous cities in the urban agglomeration. The top four GDP cities in the study area (Shenzhen, Guangzhou, Foshan and Dongguan) have the largest migration rates and relatively high epidemic severity. Most of the directions of population flow are mutual, that is, OD lines of migration appear in pairs, such as the largest migration from Guangzhou to Foshan and Foshan to Guangzhou, Shenzhen to Dongguan and Dongguan to Shenzhen. Population mobility is not always positively correlated with the severity of the epidemic. The inflow of Huizhou, Zhongshan and Zhuhai is negatively correlated with the severity of the epidemic, that is, the inflow decreases in turn, but the severity of the epidemic is serious for each. Both Jiangmen and Zhaoqing accounted for less than 5% of the total migration scale index. Jiangmen and Zhaoqing have large land areas and extremely low epidemic severity. Although Hong Kong and Macao have a high degree of economic development, people from the mainland primarily migrate to Hong Kong and Macao for work and study. The peak of the Spring Festival travel rush usually appear an outflow of people, so there was an occasional but small amount of immigration during this time, and the severity of the epidemic is thus relatively low.

**Fig 7:**
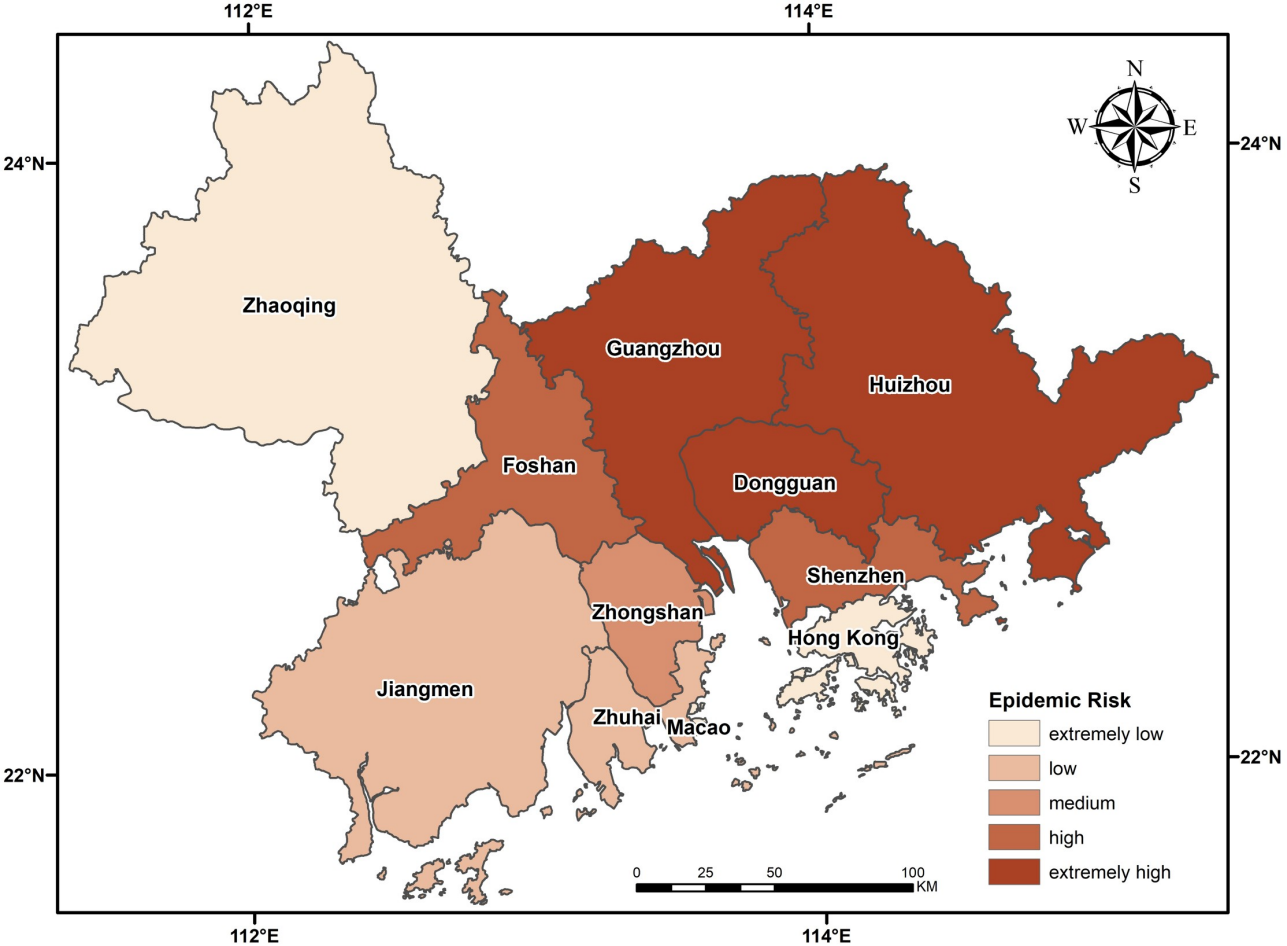


The calculated migration data and the epidemic severity index of each city were input into the epidemic transmission force model, and the epidemic risk level diagram of mutual migration from urban agglomeration was obtained. As shown in Fig 7, the overall risk of COVID-19 in the eastern part of the city cluster is higher than that in the western part, with Huizhou, Dongguan and Guangzhou as the cities with the highest risk. Huizhou has a relatively low influx and severity of COVID-19, but the high severity of COVID-19 from origin areas increases the risk of COVID-19. Foshan and Shenzhen are cities at high risk of COVID-19, and Zhongshan is at medium risk of COVID-19. The migration volume and severity of COVID-19 in these three cities are significantly higher than rates in other cities, but the the low severity of COVID-19 of origin from risk areas reduces the overall risk of COVID-19. The cities at low risk were Jiangmen, Zhuhai and Macau, with the lowest levels in Zhaoqing and Hong Kong. The proportion of both low risk and low scale of urban migration is less than 5%, and the severity of the epidemic is low and extremely low except for Zhuhai, which is a point of origin that spread across many provinces in China; however, the small proportion of migration originating from an area of risk leads to reduced risk.

### Dispersive transmission force of the urban agglomeration

**Fig 8:**
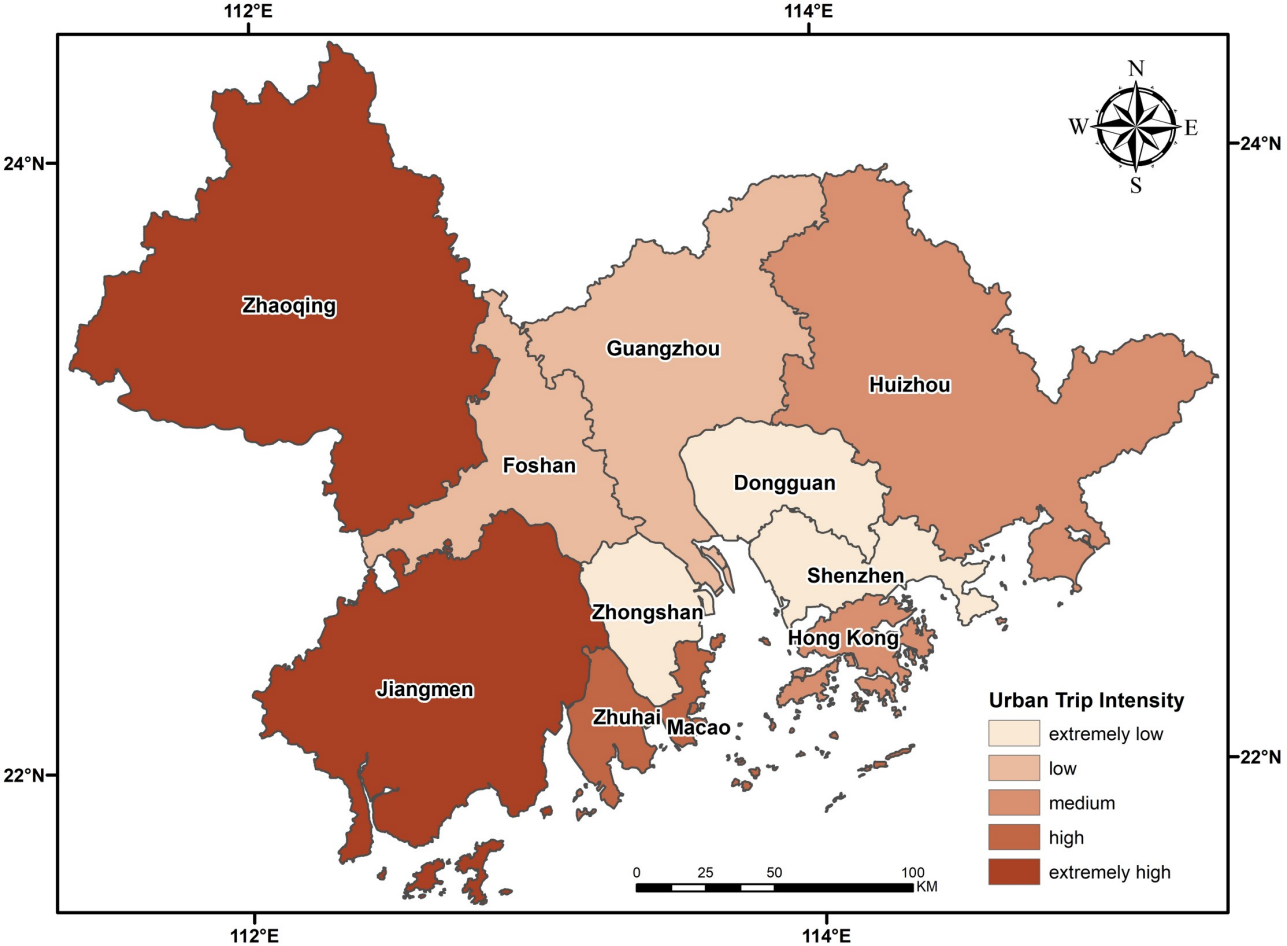


The inner-city travel intensity of each city in the urban agglomeration is calculated and graded, as shown in Fig 8. As shown in this figure, the lower the severity of the epidemic is, the higher the intensity of urban travel. Jiangmen and Zhaoqing had the highest travel intensity and the lowest epidemic severity, while Shenzhen, Zhongshan and Dongguan had the lowest travel intensity but relatively high epidemic severity. The intensity of urban travel in Foshan, Zhuhai, Hong Kong and Macao is two colors higher than the severity of the epidemic, Guangzhou is one color higher, and Huizhou is medium in both intensity and severity. To sum up, the severe epidemic areas have restricted travel within the city, and work is mostly performed at home.

**Fig 9:**
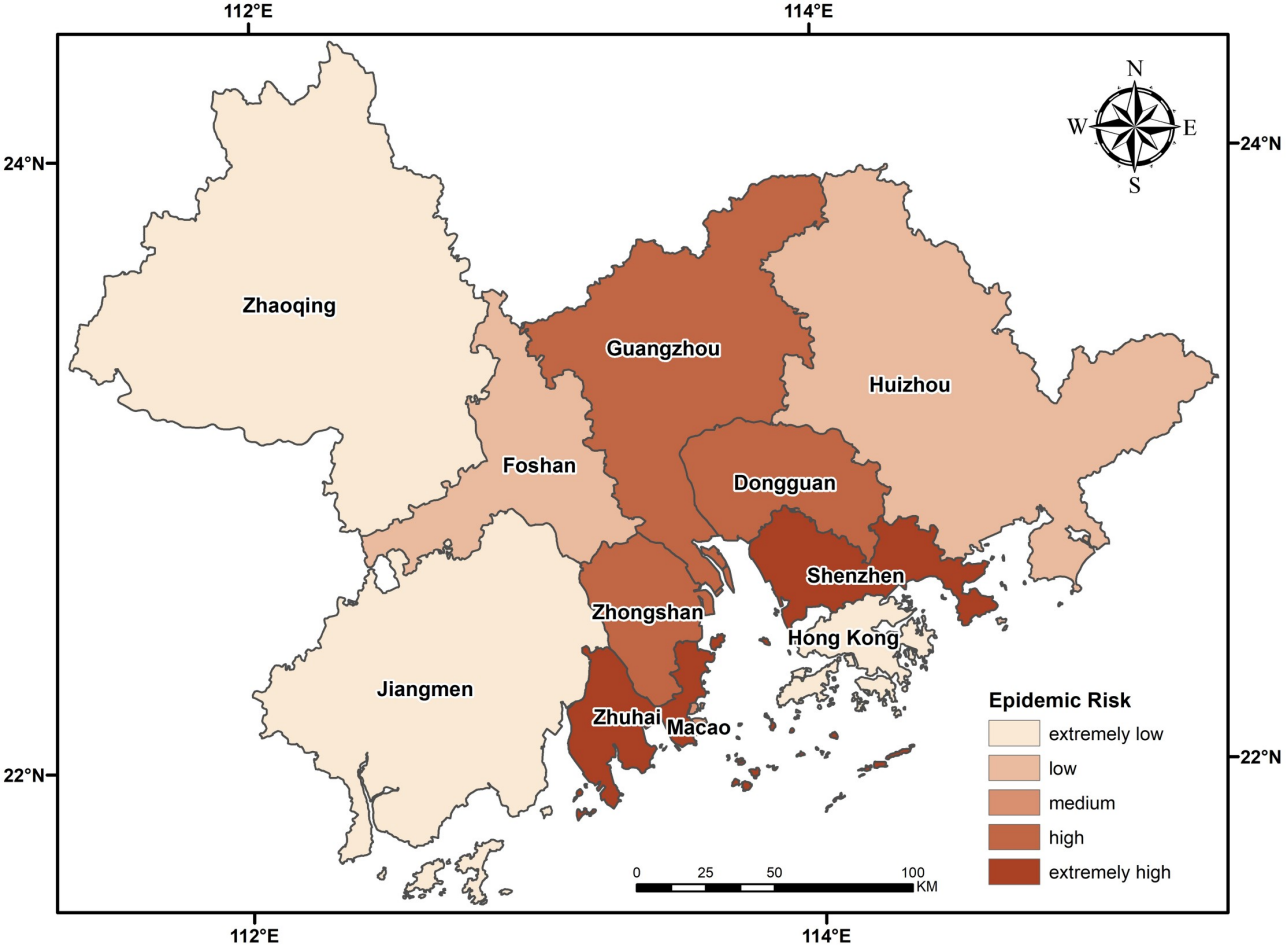


The calculated travel data and the epidemic severity force index of each city were input into the epidemic propagation force model, as shown in Fig 9. The risk of outbreak cause by the visible city travel and cities’ epidemic severity were positively correlated. Jiangmen and Zhaoqing have the lowest level of epidemic severity and disease risk; Guangzhou, Dongguan and Foshan have a high level of epidemic severity and disease risk; and Shenzhen and Zhuhai have the highest level of epidemic severity and disease risk. The disease severity and outbreak risk level in Zhongshan and Huizhou reflect the synchronization of change, because the disease severity is medium, but the disease risk is low. Although travel is restricted in severely affected areas, the risk of epidemic in these areas remains extremely high. Therefore, travel in severely affected areas still needs to be restricted to the maximum extent.

### Epidemic prevention and control pressure in urban agglomeration

**Fig 10:**
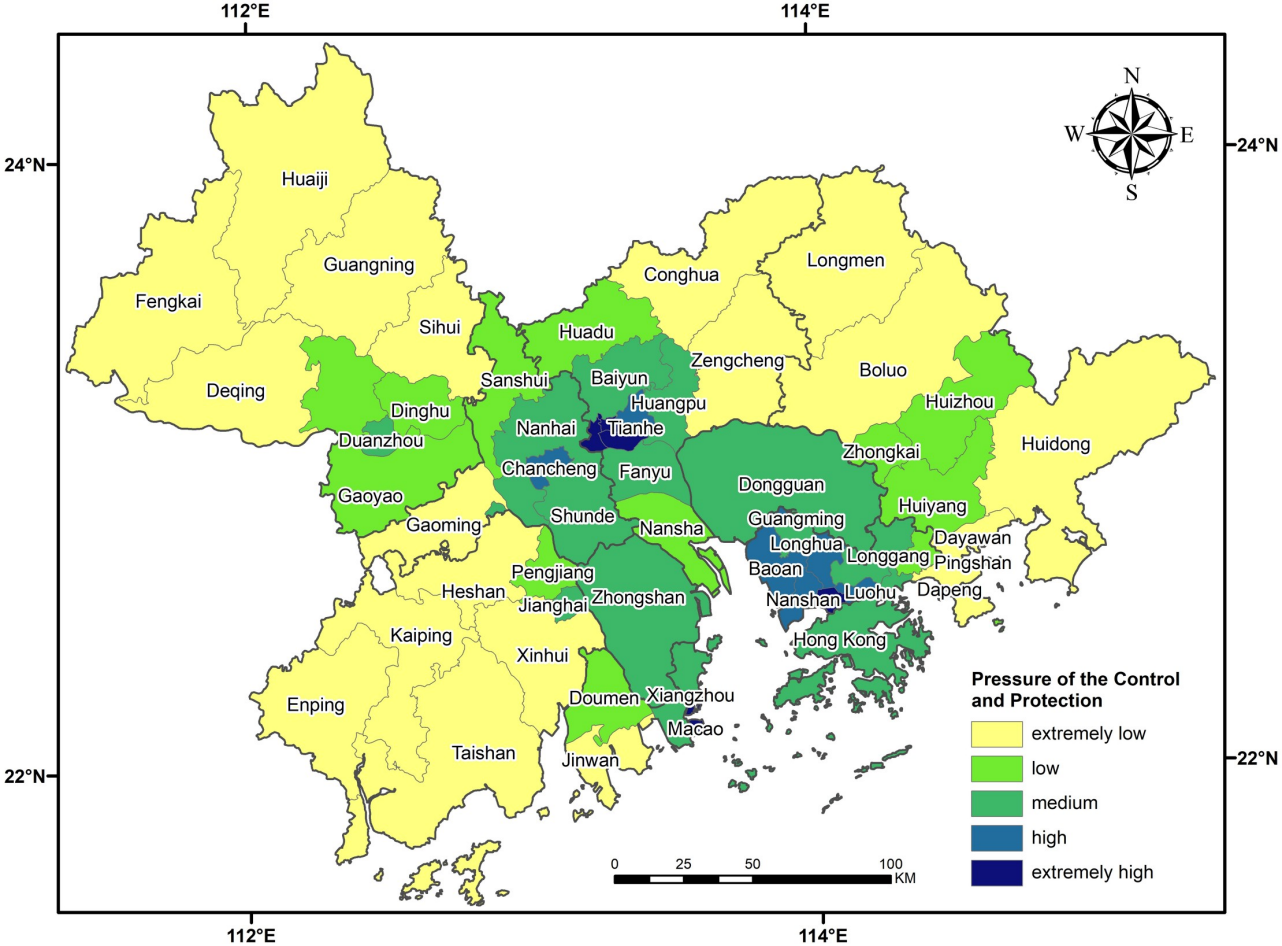


To model the prevention and control pressure to return to work and production after the outbreak of urban agglomeration from city to city, in this paper, we choose related factors concerning population density. These 4 categories of evaluation factors contain 14 subclasses of POI data, classified into population distribution, transportation, health care and life services. The evaluation factors comprise 4 categories in the district under study as the unit for all subclasses for the nuclear density analysis of POI, including the following controls: pixel unified size is 500 * 500 (m^2^), and the search radius is unified according to the type to make our data consistent with the actual data. The weight tables of 14 seeds were calculated and evaluated through factor-paired comparison and expert assignment. After all the types of nuclear density data in the research unit were homogenized, the epidemic prevention and control pressure model was established according to the values in the weight tables, as shown in Fig 10 below. As seen in Table 1, the weights of population distribution, transportation hub, medical and health care and life services decrease in turn. On the whole, the population density of the four categories is flaky and concentrated in the central part of the urban agglomeration, and the population density around it is the lowest. From a local point of view, the population density of small urban agglomeration as the center of the distributed circle decreased.

**Table 1:** The weight table of factors for the pressure rating of epidemic prevention and control.

Evaluation Factors	Factor Weights	Evaluation Factor Subclass	Condition Weights	Normalizing Weights
Population Distribution	0.43	Working Population Density	0.65	0.28
		External Passenger Flow Density	0.28	0.12
		Resident Population Density	0.07	0.03
Transportation Hub	0.29	Airport	0.42	0.12
		Railway Station	0.26	0.07
		Port	0.16	0.05
		Motor Station	0.10	0.03
		Subway Station	0.06	0.02
Medical Care	0.17	Fever Clinic	0.87	0.15
		Hospital	0.10	0.015
		Clinic	0.03	0.005
Daily Services	0.11	Bazaar	0.50	0.06
		Supermarket	0.28	0.03
		Mall	0.22	0.02

**Fig 11:**
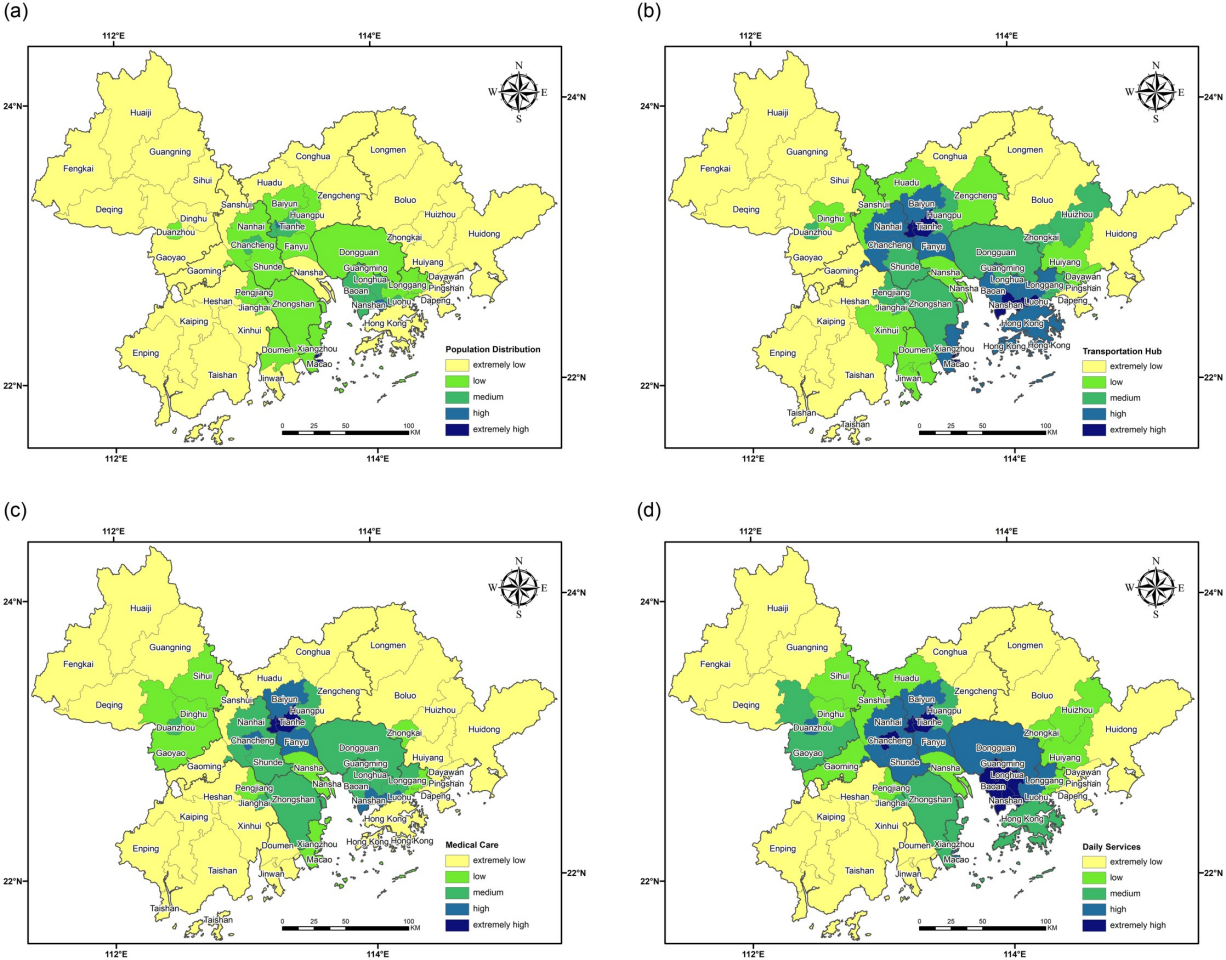


1. Population distribution is divided into working population, migrant population and resident population density, with the highest weight indicating that population distribution is the most important among the four elements. The population distribution density is low on the whole, and there are relatively high-density and extreme high-density concentration areas. The concentration points in the Macao Special Administrative Region, Futian District in Shenzhen and Yuexiu District in Guangzhou gradually decrease in the surrounding areas (Fig 11(a)).
2. Transportation hubs, including airports, ports and subway stations, are factors with higher weight and population density. The density concentration areas of transportation hubs are located mainly in the Macao Special Administrative Region, Luohu District, Nanshan District and Futian District in Shenzhen, and Haizhu District, Tianhe District, Yuexiu District and Liwan District in Guangzhou. In the longitudinal comparison of the four factors, Hong Kong has the highest density concentration, primarily because it is located in the "keypoint of traffic" of the Pearl River and the South China Sea, with a dense and convenient transportation network and the most ports (Fig 11(b)).
3. Health includes clinics, clinics and hospitals. The highest density of medical services and health in the region’s is focused on Haizhu District, Tianhe District, Yuexiu District and Liwan District. The main reason for this density is that Canton enjoys a high-level medical reputation. Because of the influence of the outbreak, the concentration of the large number of new fever outpatient service areas means that population density after standardization is the largest in this region (Fig 11(c)).
4. The life services category includes three smaller subcategories, namely, bazaars, supermarkets and shopping malls. Life services provide people with material necessities of life and spiritual nourishment. Therefore, the most concentrated areas with high density and extremely high density cover 11 areas in four prefecture-level cities: Guangming District in Shenzhen; Baoan District, Longhua District, Nanshan District and Futian District, Yuexiu District, Liwan District, Haizhu District and Tianhe District in Guangzhou; Chancheng District in Foshan; and Duanzhou District in Shiuhing. When the "regional groups" of these four prefecture-level cities with high density are taken as the center, the degree of population density concentration gradually decreases (Fig 11(d)).

The density of transportation hubs, medical and health services and living services is dispersed in patches; the numerous aggregation areas exist because people cannot live without clothing, transportation, housing and food. The higher the levels of urban economic development and per capita consumption are, the larger the proportion in the weighted grading map. The overall pressure of prevention and control is roughly similar to the weight classification of the four evaluation factors, and the concentrated density area is distributed in the center of the Guangdong-Hong Kong-Macao Greater Bay Area. The highest levels of prevention and control pressure are found in the Macao Special Administrative Region; Futian District in Shenzhen; and Haizhu District, Liwan District and Yuexiu District in Guangzhou, which have increased the pressure due to the large number of migrant workers in these areas. The high-pressure points for prevention and control are Duanzhou District in Shiuhing, Chancheng District in Foshan, Tianhe District in Canton, and Longhua District, Baoan District, Luohu District and Nanshan District in Shenzhen. The districts and counties with the high and extremely high levels of prevention and control pressure are taken as the core of the region, and the further an area is from the core cities, the lower its level of prevention and control pressure.

## Conclusions

Taking a world-class city cluster, the Guangdong-Hong Kong-Macao Greater Bay Area, as the research area, this study used official migration data and Baidu migration regions, as well as relevant POI data, combined with OD flow and kernel density analysis method, to model a prevention and control of epidemic spread force index and a pressure rating model. The study explored the level of risk posed by population flow to urban agglomerations and the level of pressure to return to work after a comeback in the area of the urban agglomeration. The main conclusions are as follows:

1. In the analysis of migration from Wuhan to urban agglomerations, the correlation coefficient between the scale index of the migration from Wuhan to urban agglomerations and the severity of the epidemic is 0.98, which shows a significant correlation that is basically consistent with the assertion that higher the migration volume is, the higher the epidemic severity. With Guangzhou and Shenzhen as the axis, the severity of the epidemic gradually decreases to the east and west.
2. In the analysis of the transmission force of urban agglomeration, except for Hong Kong and Macao, the cities with migration to the urban agglomeration are mainly affected by the geographical proximity and the population base of the migration source. In addition, the Pearl River Delta and Yangtze River Delta regions with higher GDP contributed more to the inflow of Hong Kong and Macao. The classification of epidemic risk depends mainly on the migration scale index and the epidemic severity. Shenzhen, Guangzhou and Tungkun are all located in the Pearl River Estuary Delta and are important hubs of the Beijing-Kowloon Railway. Convenient transportation, advantageous location and prosperous economy have greatly increased the inflow and outflow of population in these three regions, leading to the highest epidemic risk. In general, the higher the migration scale index and the epidemic severity level are, the higher the epidemic risk level at the destination.
3. In the analysis of the imported transmission force among cities in the urban agglomeration, population mobility tends to be into the economically developed cities, but the classification of epidemic risk level is not related simply to geographical proximity and regional economic development. In contrast, the number of confirmed cases and the total local registered population are also decisive factors. The high-risk areas were distributed in the northeastern regions of the study area, showing a stepwise decrease from east to west, and from north to south.
4. In the propagation force analysis of internal diffusivity inside of the agglomeration, because those cities with the higher disease severity limit mass travel activities, so the city population flow are negatively associated with the disease severity of each city. However, areas with economic dynamism and a high degree of openness to areas outside the city, such as Zhuhai and Shenzhen, face a greater risk of outbreaks. The severity of the epidemic is basically consistent with the classification of epidemic risk in each city. Cities with high epidemic severity are generally at high epidemic risk, and population mobility still needs to be reduced in these areas. The resumption of school and work needs to be carried out in batches according to the epidemic risk in each city.
5. In the analysis of the epidemic prevention and control pressure in the urban agglomeration after the resumption of work and production, the population density of population distribution, transportation hubs, medical and health care and life services showed patchy distribution, and the population was mainly concentrated in the northwestern and central areas of the Guangdong-Hong Kong-Macao Greater Bay Area. Among these factors, population distribution has the highest weight, transportation hubs and life services are necessary services in daily life, and population density is the largest. The degree of population concentration in medical and health care gradually increased with the epidemic severity. The final pressure level of prevention and control is obtained by combining various factors. Haizhu District, Liwan District and Futian District of Shenzhen are taken as the core of a circular region; the further an area is from the core city, the lower the pressure level of prevention and control is.

## Discussion

To sum up, this paper aims to obtain an epidemic prevention and control pressure index through a linkage analysis tracking the movement of people and the actual confirmed case data in the Guangdong-Hong Kong-Macao Greater Bay Area, a typical urban agglomeration region. The above pressure index examines movement within the Guangdong area and could also be used to examine other aspects of the global epidemic. The combination of actual case and diagnosis data by precise location and amount of accurate data used to obtain an outbreak "map" allows for the creation of a pressure index layer overlay indicating prevention and control information, which can greatly improve the precision and efficiency of epidemic control. The above results combine the latest geographic information technology with practical work and use science and technology to improve the efficiency of disease prevention and control work. The results can be applied at a small scale to the prevention and control efforts by rural and urban internal, street, and community personnel, as well as facility and unattended supply directions. They can also be of great use to municipal, provincial and even national efforts to return to work, providing a reference for regional planning and production. Especially for such administrative departments as government agencies, sufficient and accurate information reference provide the basis for further conquering the epidemic. Even in the face of future major events similar to this COVID-19 outbreak, the research model of this paper can be applied to the correlation study of event carrier flow and the emergence of final results, and different types of stress indexes can be proposed to provide reference bases with which relevant departments and the public can solve problems. In addition, this paper mainly discusses the transmission force and the pressure of prevention and control of the epidemic, which is mainly reflected in the changing trend of transmission, so there is lag in diagnosis, and time series and other methods will be used in subsequent studies to improve the analysis methods and models.

## Supporting information

S1 Table China city code comparison table. The table reports the codes for each province and city in China. The purpose is to compare and illustrate the city codes in subsequent tables.
(XLSX)

S2 Table Urban migration data. The table is composed of eleven sheets. The eleven sheet names are compared with the eleven cities in the urban agglomeration, which is the original data of the migration scale of the eleven cities.
(XLSX)

S3 Table Intra-city travel data. The table is the original data of daily intra-city travel intensity of eleven cities in the urban agglomeration during the study period.
(XLSX)

S4 Table Urban population and epidemic data. The table reports the registered population of the cities relevant to the study, as well as the number of confirmed cases in that city at the time of the study.
(XLSX)

S5 Table Poi data. The table is composed of eleven sheets. Three sheet names are the major class names of the POI, where h1 represents the big class of POI and h2 is a subclass of h1.
(XLSX)

## References

[1] Liu T, Hu J, Xiao J, He G, Kang M, Rong Z, et al. Time-varying transmission dynamics of Novel Coronavirus Pneumonia in China J. bioRxiv 2020.

[2] Wu J.T Leung K Leung G.M. Now casting and forecasting the potential domestic and international spread of the 2019-nCoV outbreak originating in Wuhan China: A modelling study. J. Lancet. 2020;395(1): 689–697. 10.1016/S0140-6736(20)30260-9 PMC7159271 32014114

[3] Chan J.F, Yuan S, Kok K.H, To K.K, Chu H, Yang J, et al. A familial cluster of pneumonia associated with the 2019 novel coronavirus indicating person-to-person transmission: A study of a family cluster. Lancet. 2020;395:514–523. 10.1016/S0140-6736(20)30154-9 31986261 PMC7159286

[4] Liu Y, Gayle A.A, Wilder-Smith A, Rocklöv J. There productive number of COVID19 is higher compared to SARS coronavirus. J. Travel Med. 2020,27. 10.1093/jtm/taaa021 PMC7074654 32052846

[5] Huang C, Wang Y, Li X, Ren L, Zhao J, Hu Y, et al. Clinical features of patients infected with 2019 novel coronavirus in Wuhan China. J. Lancet.2020;395(23):497–506. 10.1016/S0140-6736(20)30183-5 PMC7159299 31986264

[6] Malik Y.S, Sircar S, Emerging novel coronavirus (2019-nCoV)-current scenario evolutionary perspective based on genome analysis and recent developments. J. The veterinary quarterly. Lancet. 2020;40(1):68–76. 10.1080/01652176.2020.1727993 32036774 PMC7054940

[7] Yang Y.S, Peng F.J. The deadly coronaviruses: The 2003 SARS pandemic and the 2020 novel coronavirus epidemic in China. J. Journal of autoimmunity 109. 10.1016/j.jaut.2020.102434 PMC7126544 32143990

[8] Chen S, Xu W. The first case of novel Coronavirus was confirmed in Guangdong. http://www.bjnews.com.cn/news/2020/01/20/676734.html. (accessed 20 January 2020).

[9] Li GJ, Li YK Hong Kong may have its first confirmed case of COVID-19. http://finance.sina.com.cn/stock/hkstock/hkstocknews/2020-01-22/doc-iihnzahk5816053.shtml (accessed on 22 January 2020).

[10] Ren L.J. The first confirmed case of novel Coronavirus infection occurred in Macau. http://www.chinanews.com/ga/2020/01-22/9066991.shtml (accessed on 22 January 2020).

[11] Lee E.C, Asher J.M, Goldlust S, Kraemer J.D, Lawson A.B, Bansal S. Mindthescales: Harnessingspatial big data for infectious disease surveillance and inference. J. Infect. Dis. 2016;214 S409–S413. 10.1093/infdis/jiw344 28830109 PMC5144899

[12] Zheng P, Wang R, Liu L.B, Wu H. (2020). Exploring Urban Spatial Features of COVID-19 Transmission in Wuhan Based on Social Media Data. ISPRS International Journal of Geo-Information. 9. 402. 10.3390/ijgi9060402

[13] Bansal S, Chowell G, Simonsen L, Vespignani A, Viboud C. Big data for infectious disease surveillance and modeling. J. Infect. Dis. 2016 214 S375–S379. 10.1093/infdis/jiw400 28830113 PMC5181547

[14] Zian Z, Shi Z.J. Preliminary estimation of the novel coronavirus disease (COVID-19) cases in Iran: A modelling analysis based on overseas cases and air travel data. International Journal of Infectious Diseases. 2020;94(3):29–31. 32171951 10.1016/j.ijid.2020.03.019 PMC7194910

[15] Sarkodie S.A, Owusu P.A, Investigating the cases of novel coronavirus disease (COVID-19) in China using dynamic statistical techniques. Heliyon 6(4):e03747. 10.1016/j.heliyon.2020.e03747 32289090 PMC7128585

[16] Liu Z, Qian J.l, Du Y.Y, Wang N, Yi J.W, Sun Y.R, et al. Multi-levelSpatial Distribution Estimation Model of the Inter-regional Migrant Population Using Multi-source Spatio-temporal Big Data: A Case Study of Migrants from Wuhan during the Spread of COVID-19. J. Journal of Geo-information Science. 2020;22(2): 147–160.

[17] Mei W.J, Liu Z, Zhu J.Y, Du L. Extreme IR Model for COVID -19 Real-Time Forecasting. J. Journal of University of Electronic Science and Technology of China. 2020;49(3): 362–368.

[18] Zhu R.J, Tang S.H, Liu T.T, Guo Y, Dong S.S, Cheng Y, et al. COVID-19 epidemic prediction based on improved SIR model and the impact of prevention and control on epidemic development. J. Journal of Shaanxi Normal University (Natural Science Edition). 2020;(03):38–43.

[19] Xu X.k, Wen C, Zhang G.Y, Sun H.C, Liu B, Wang X.W. The Geographical Destination Distribution and Effect of Outflow Population of Wuhan When the Outbreak of COVID-19. J. Journal of University of Electronic Science and Technology of China. 2020;49(3): 324–329.

[20] Hua J, Shaw R. Corona Virus (COVID-19) "Infodemic" and Emerging Issues through a Data Lens: The Case of China. Int. J. Environ. Res. Public Health. 2020;(17):2309. 10.3390/ijerph17072309 32235433 PMC7177854

[21] Shaw R, Kim Y.K, Hua J. Governance technology and citizen behavior in pandemic: Lessons from COVID-19 in East Asia. Prog. J. Disaster Sci. 2020 10.1016/j.pdisas.2020.100090 PMC7194878 34171010

[22] Wiki. 2020 Coronavirus Pandemic in Japan. 2020. https://en.wikipedia.org/wiki/2020_coronavirus_pandemic_in_Japan (accessed on 15 April 2020).

[23] Gao S, Rao J, Kang Y, Liang Y, Kruse J. Mapping county-level mobility pattern changes in the United States in response to COVID-19. J. Sigspatial Spec. 2020;16(25):16–26.

[24] Huang R, Liu M, Ding Y. Spatial-temporal distribution of COVID-19 in China and its prediction: A data-driven modeling analysis. J. Infect. Dev. Ctries. 2020;14(1):246–253. 10.3855/jidc.12585 32235084

[25] Guangdong Provincial Bureau of Statistics. Statistical Yearbook of Guangdong Province [M]. BEIJING: China Statistics Press, 2019.

[26] Hong Kong Bureau of Statistics. Hong Kong Statistical Yearbook [M]. BEIJING: China Statistics Publishing House, 2019.

[27] Statistical and Census Bureau of the Government of the Macao Special Administrative Region. 2019. https://www.dsec.gov.mo/zh-MO/

[28] National Health Commission of the Peoples Republic of China. http://www.nhc.gov.cn/zhuz/index.shtml

[29] Baidu Map Open Platform—Web Service API. http://lbsyun.baidu.com/index.php?title=webapi/guide/webservice-placeapi

[30] Kang D, Choi H, Kim J.-H, Choi J. Spatial epidemic dynamics of the COVID-19 outbreak in China. Int. J. Infect. Dis. 2020;(94):96–102. 10.1016/j.ijid.2020.03.076 32251789 PMC7194591

[31] Wu Q.Y, Su K.Y, Zou Z.J. Spatial and temporal analysis of bus passenger flow based on massive smart card data. J. Journal of Geo- information Science. 2018;20(5):647–655.

[32] Xin R, Ai T.H, Yang W, et al. A new network voronoi diagram considering the OD point density of taxi and visual analysis of OD flow. J. Journal of Geo- information Science. 2015;17(10): 1187–1195.

[33] Zhou Z, Meng L, Tang C, et al. Visual abstraction of large scale geospatial origin- destination movement data. J. IEEE transactions on visualization and computer graphics. 2018;25(1):43–53. 10.1109/TVCG.2018.2864503 30130199

[34] Guo D, Zhu X, Jin H. et al. Discovering spatial patterns in origin- destination mobility data. J. Transactions in GIS. 2012;16(3):411–429.

[35] Yao X, Zhu D, Gao Y, et al. A stepwise spatio- Temporal flow clustering method for discovering mobility trends. J. IEEEACCESS. 2018;6:44666–44675.

[36] Zhao W.F, Li Q.Q, Li B.J, Extracting Hierarchical Landmarks from Urban POI Data. J. Journal of Remote Sensing. 2011;15(5): 973–988.

[37] Zhang L. Research on POI Classification Standard. J. Bulletin of Surveying and Mapping. 2012;(10): 8284.

[38] Yu W.H, Ai T.H, The Visualization and Analysis of POI Features under Network Space Supported by Kernel Density Estimation. J. Acta Geodaetica et Carto-graphica Sinica.2015;44 (1): 82–90.

[39] Yu W.H, Ai T.H, Yang M, Liu J.P. Detecting “Hot Spots"of Facility POIs Based on Kernel Density Estimation and Spatial Autocorrelation Technique. J. Geomatics and Information Science of Wuhan University. 2016;41(2): 221–227.

[40] Dou Z. Spatial Clustering Algorithm on Urban Function Oriented Zone D. Sichuan: Sichuan Normal University 2010.

